# Identifying diabetics in Medicare claims and survey data: implications for health services research

**DOI:** 10.1186/1472-6963-14-150

**Published:** 2014-04-03

**Authors:** Joseph W Sakshaug, David R Weir, Lauren H Nicholas

**Affiliations:** 1 Institute for Social Research, University of Michigan, 426 Thompson Street, Ann Arbor, MI, 48104, USA; 2 Institute for Employment Research, Regensburger Str. 104, Nuremberg, 90478, Germany; 3 Health Policy & Management, Johns Hopkins School of Public Health, 624 N. Broadway, Hampton House 450, Baltimore, MD, 21205, USA

**Keywords:** Diabetes, Survey data, Medicare claims, Chronic condition warehouse

## Abstract

**Background:**

Diabetes health services research often utilizes secondary data sources, including survey self-report and Medicare claims, to identify and study the diabetic population, but disagreement exists between these two data sources. We assessed agreement between the Chronic Condition Warehouse diabetes algorithm for Medicare claims and self-report measures of diabetes. Differences in healthcare utilization outcomes under each diabetes definition were also explored.

**Methods:**

Claims data from the Medicare Beneficiary Annual Summary File were linked to survey and blood data collected from the 2006 Health and Retirement Study. A Hemoglobin A1c reading, collected on 2,028 respondents, was used to reconcile discrepancies between the self-report and Medicare claims measures of diabetes. T-tests were used to assess differences in healthcare utilization outcomes for each diabetes measure.

**Results:**

The Chronic Condition Warehouse (CCW) algorithm yielded a higher rate of diabetes than respondent self-reports (27.3 vs. 21.2, *p* < 0.05). A1c levels of discordant claims-based diabetics suggest that these patients are not diabetic, however, they have high rates of healthcare spending and utilization similar to diabetics.

**Conclusions:**

Concordance between A1c and self-reports was higher than for A1c and the CCW algorithm. Accuracy of self-reports was superior to the CCW algorithm. False positives in the claims data have similar utilization profiles to diabetics, suggesting minimal bias in some types of claims-based analyses, though researchers should consider sensitivity analysis across definitions for health services research.

## Background

Diabetes has become one of the most common and expensive medical conditions amongst older adults. Nearly 25% of all adults ages 60 and older have diabetes in the United States [1] and this group accounts for over half of the more than $100 billion in healthcare expenditures attributable to this disease [2]. Reducing the disease and economic burden of diabetes has been a long-standing goal in health policy.

Carefully designed analyses of secondary datasets, including Medicare claims data, can contribute to diabetes health services research. The strength of this design is critically dependent on being able to identify diabetic patients in datasets. Utilization-based algorithms are frequently used to identify chronic disease cohorts in national Medicare data and other administrative datasets. However, concordance between disease status assessed by these algorithms and patient self-reports can be low, raising concern about the bias associated with research designs relying on non-clinical data [3,4].

A diabetes diagnosis requires clinical measures of Hemoglobin A1c (the gold standard since 2010), a measure of blood sugar control over the past 60–90 days, or fasting blood sugar [5]. However, obtaining clinical measures of diabetes status for health services research is rare, and most researchers rely on more subjective measures, such as self-reports or claims-based diagnoses to identify the diabetic population. Both measures have strengths and limitations.

Self-reports are commonly used to assess diabetes prevalence in survey data when biological measures are unavailable [6-9]. Self-reports are believed to underestimate diabetes prevalence because they depend on a patient seeing a doctor to receive a clinical diagnosis and correctly reporting when asked. The standard approach in surveys with biomarkers is to take all self-reports as true cases and add in the people who self-report “no” but test above the diagnostic threshold. The National Health and Nutrition Examination Survey (NHANES) is the most common data source used for studying the population of undiagnosed diabetics. The latest published NHANES estimates for the 65 and older population for years 2003–2006 indicate a diabetes rate of 21.1% based on the sum of self-reported diabetics (17.7%) and self-reported non-diabetics with an A1c reading greater than or equal to 6.5 (3.5%) [10].

Claims-based diagnoses may be obtained from patient billing records, hospital discharge abstracts, and physician data and are usually based on algorithms that may or may not match actual diagnoses found in medical records [11]. The Center for Medicare and Medicaid Services’ Chronic Condition Warehouse (CCW) algorithm is the primary diabetes algorithm used in Medicare claims-based research as it is included in their research files and many users are likely to implement it given inclusion in the Beneficiary Annual Summary File. The CCW algorithm defines a diabetic as someone who has had at least one inpatient, skilled nursing or home health visit or at least two outpatient visits with a diabetes-related ICD-9 code during a two year period [12]. This definition was observed to be adequately sensitive (≥70%) and reliable (kappa ≥ 0.80) [13]. Other studies of administrative claims-based measures of diabetes status have yielded varying sensitivities ranging from 64% to 87% when validated against different benchmarks, including laboratory data, medical records, and self-reports [4,14-16].

Investigating the quality of survey self-report and claims-based diabetes measures is important because data users rely on these measures in health services research to produce population-based estimates of the diabetes population. These estimates often have important policy implications and any inaccuracies can lead to incorrect inferences. For example, overestimation of the diabetes population may lead to misleading conclusions about the quality of diabetes care as the false positives may be seen as not receiving adequate amounts of care. Conversely, underestimation of the diabetes population may lead to conclusions that understate the prevalence of the disease and the economic burden of the disease.

This paper provides some insight as to the accuracy of self-report and CCW claims-based diabetes measures and their implications for health services research. In it, we use nationally representative survey data linked to Medicare claims and measured Hemoglobin A1c levels from an in-person blood draw to compare the accuracy of the self-report and CCW measures relative to the A1c reading. Furthermore, we examine discrepancies between the two measures and compare commonly used healthcare utilization outcomes across each of the diabetes definitions.

## Methods

We utilize the 2006 wave of the Health and Retirement Study (HRS) [17]. The HRS is a nationally representative longitudinal study of Americans over the age of 50. In 2006, HRS began collecting several physical measurements, including biologic specimens: saliva and dried blood spots. Here we focus on the collection of blood spots and measured Hemoglobin A1c to validate and understand discrepancies in self-reported and claims-based diabetes status, which was obtained for 83 percent of the biomarker subsample who consented to the blood draw.

Medicare-eligible HRS respondents were asked for permission to link their Medicare records from the Centers for Medicare and Medicaid Services (CMS). HRS data and records from the 2006 Medicare Beneficiary Annual Summary Files were linked for 88 percent of respondents who consented to the linkage. Consent rates were higher for younger, wealthier, Hispanic, and non-white respondents. Diabetes prevalence is higher amongst minorities and the younger-old [2], suggesting that the self-selected subsample represents persons who are more likely to be diabetic.

Three measures of diabetes status were utilized: survey self-reports (“Has a doctor ever told you that you have diabetes or high blood sugar?”), indication of diabetes based on the Chronic Condition Warehouse (CCW) algorithm, and a Hemoglobin A1c score of 6.5 or higher [5].

Four healthcare utilization outcomes were extracted from the Medicare claims and used to assess differences between the diabetes definitions. The first three outcomes (total Medicare reimbursement, number of office visits, and number of hospitalizations in 2006) relate to general healthcare usage. The last outcome, the number of A1c tests ordered between years 2002–2005, is used as an indicator of quality of diabetes care, a commonly used measure in quality of care studies [18-21].

We restrict the sample to persons aged 65 years and older who were Medicare-eligible at the time of interview (n = 11,354). The sample was further restricted to individuals randomly selected for biomarker collection (n = 5,784), of which 3,820 completed the blood draw and received a valid A1c score. Of these cases, Medicare records were linked to 3,389 respondents. Beneficiaries who were not continuously enrolled in fee-for-service Medicare were excluded (n = 1,185) along with potential users of Veterans Affairs (VA) services to ensure that all health care utilization was reflected in the claims (n = 176). The resulting analytic sample consisted of 2,028 respondents.

Concordance between the self-report and claims data was assessed using t-tests and two-way tables. Mean A1c levels and utilization outcomes are reported for the concordant and discordant cases. All analyses were performed using appropriate survey procedures in SAS 9.1.3 inside a secure data enclave at the Institute for Social Research in Ann Arbor, Michigan. This study was exempt from Institutional Review Board oversight.

## Results

First, to assess the comparability of our analytic sample we compare diabetes estimates from the linked HRS-Medicare-A1c sample with those from three national samples: the 2005–2006 National Health and Nutrition Examination Survey (NHANES), the linked HRS-A1c only sample, and the linked HRS-Medicare only sample. All samples are restricted to the Medicare-eligible population aged 65 and older. Table 1 shows that the percentage of self-reported diabetics (Table 1) (21.1; 95% CI: 19.2-23.2), clinical diabetics with A1c reading ≥ 6.5 (12.6; 10.7-14.4), and undiagnosed diabetics (4.4; 95% CI: 3.2-5.6) in the linked HRS-Medicare-A1c sample are statistically comparable to the corresponding estimates obtained from the other three samples, suggesting consistency across the sources and minimal bias in using the linked HRS-Medicare-A1c sample.

**Table 1:** Prevalence of Diabetes amongst Adults ages 65 and older in Three National Samples

	**NHANES**	**Linked HRS/A1c**	**Linked HRS/Medicare**	**Linked HRS/Medicare/A1c**
Self-report	19.1% (16.6-21.6)	21.3% (19.8-22.8)	21.7% (20.4-23.0)	21.2% (19.2-23.2)
Hemoglobin A1c ≥ 6.5	12.2% (10.1-14.3)	13.0% (11.8-14.1)	--	12.6% (10.7-14.4)
Undiagnosed	3.8% (1.8-5.7)	4.2% (3.3-5.2)	--	4.4% (3.2-5.6)
Self-report + undiagnosed	22.9%	25.5%	--	25.6%
Medicare claims	--	--	28.7% (27.1-30.2)	27.3% (25.3-29.2)
Sample size	1,066	3,817	6,101	2,028

Parenthetical entries are 95% confidence intervals.

Undiagnosed diabetes refers to the percentage of cases who self-reported not ever receiving a diabetes diagnosis but met the clinical threshold for diabetes (Ha1c ≥ 6.5).

Sources: 2006 Health and Retirement Study (HRS); 2006 Medicare Beneficiary Annual Summary File; 2005–2006 National Health and Nutrition Examination Survey (NHANES).

The CCW claims-based algorithm yields a significantly higher percentage of diabetes compared to self-reports (Claims: 27.3; SRs: 21.2; *p* < 0.05). However, the overall percentage of diabetes (based on the sum of self-reported and undiagnosed diabetics) obtained from the survey data (25.6) is statistically indiscernible from the claims-based estimate (27.3; 95% CI: 25.3-29.2).

The next analysis examines the two-way agreement between self-report and the claims-based diabetes definitions (Table 2). About 19.5 percent (or n = 406) of respondents are classified as diabetic based on both self-report and claims data and 71.1 percent (or n = 1,426) are classified as non-diabetics in both data sources. The remaining 9.3% of the sample (n = 196) yield a conflicting a result between the two measures. Most of the discordant cases (82.7 percent; n = 162, 7.7 percent of total sample) are flagged as diabetic in the claims data. Discordant claims-based diabetics tend to be older and self-report better health status compared to concordant diabetics (Table 3).

**Table 2:** Cross-classification of self-report and claims-based diabetes status

	**Medicare claims (%)**	
	Not diabetic	Diabetic
Self-report (%)		
Not diabetic	71.1 (1426)	7.7 (162)
Diabetic	1.6 (34)	19.5 (406)

Chi-square test statistic: 737.7; *p* < 0.05.

Cohen’s Kappa statistic: 0.74.

Parenthetical entries are sample sizes.

Claims-based diabetics are defined as those who have ever triggered the CCW definition for diabetes.

Sources: 2006 Health and Retirement Study (HRS); 2006 Medicare Beneficiary Annual Summary File.

**Table 3:** Percent of Medicare beneficiaries aged 65 and older, by Discordant (Claims) and Concordant Diabetes Classification Group and characteristics

	**Claims-based diabetics (Discordant)**	**Concordant diabetics (Self-report and claims)**
Age		
65–74	34.0 (3.7; 65)*	50.8 (2.7; 230)*
75–84	48.3 (4.3; 72)	39.5 (2.5; 148)
85+	17.8 (3.3; 25)*	9.7 (1.7; 28)*
Gender		
Male	39.5 (4.2; 62)	45.1 (2.1; 184)
Ethnicity		
Hispanic	4.4 (1.7; 9)	7.0 (1.8; 34)
Race		
Non-White	13.8 (2.8; 31)	10.2 (1.5; 58)
HH income		
(quartiles of SR & Claims group)		
Q1 ($15,827 or less)	30.1 (3.8; 50)	26.9 (2.7; 102)
Q2 ($15,828-$30,576)	21.7 (4.0; 33)	24.4 (2.3; 100)
Q3 ($30,577-$49,438)	22.0 (4.5; 37)	23.7 (1.8; 102);
Q4 ($49,439 or more)	26.3 (3.1; 42)	25.0 (2.6; 102)
Health rating	29.9 (3.2; 52)*	21.8 (2.6; 91)*
Excellent/VG	36.7 (3.4; 59)	35.5 (2.7; 144)
Good	33.5 (3.8; 51)	42.8 (4.1; 171)
Fair/Poor		
Moderate activity	45.8 (3.5; 72)	44.4 (2.5; 175)
Hardly ever/never		
Number of diseases	14.8 (2.7; 22)	9.8 (1.8; 40)
0	41.4 (3.4; 71)	37.5 (2.2; 163)
1	24.5 (3.9; 42)	31.5 (1.7; 124)
2	19.3 (3.6; 27)	21.2 (2.4; 79)
3+		
Self-Reported Memory Rating		
Excellent/Very good	16.7 (2.8; 26)	20.3 (2.0; 82)
Good	43.4 (3.7; 71)	44.2 (2.2; 173)
Fair/Poor	39.9 (3.1; 65)	35.5 (3.0; 151)
Cognitive Functioning		
(TICS Score)		
0–8	23.1 (4.3; 39)	18.1 (1.9; 68)
9	21.4 (3.7; 35)	27.4 (2.2; 111)
10	55.5 (5.1; 88)	54.6 (2.6; 227)
Total number of flagged diabetics	162	406

*Notes:* Standard errors and sample sizes are in parentheses.

Column percentages may not always add to 100 due to rounding error.

*Difference between self-report estimate and claims estimate is statistically significant *p* < 0.05.

Source*:* 2006 Health and Retirement Study; 2006 Medicare Beneficiary Annual Summary File.

Clinical classifications based on A1c readings are shown in Table 4. Not surprisingly, concordant diabetics (CDs) and concordant non-diabetics (CNDs), respectively, yield the highest and lowest percentage of clinical diabetes (A1c ≥ 6.5), respectively (CDs: 45.7; CNDs: 3.5) among the four possible agreement groups. Among the discordant cases, self-reported diabetics yield a significantly higher mean A1c reading (6.32 vs. 5.86), a higher percentage of clinical diabetes (30.5 vs. 12.4), and a lower percentage of A1c readings less than 6.0 (42.8 vs. 63.6) relative to the CCW-based diabetics (all comparisons yield *p*-value < 0.05).

**Table 4:** Hemoglobin A1c estimates by self-report and chronic condition warehouse diabetes status

**Clinical measures**	**Self-report, yes; Claims, yes**	**Self-report, yes; Claims, no**	**Self-report, no; Claims, yes**	**Self-report, no; Claims, no**
Hemoglobin A1c (mean)	6.60 (0.05)	6.32 (0.03)	5.86 (0.04)	5.64 (0.02)
Hemoglobin A1c ≥ 6.5 (%)	45.7 (2.4)	30.5 (0.5)	12.4 (2.2)	3.5 (0.6)
Hemoglobin A1c < 6.0 (%)	24.8 (2.5)	42.8 (2.9)	63.6 (2.6)	80.8 (1.4)
Sample size	406	34	162	1426

Parenthetical entries are standard errors.

Source*:* 2006 Health and Retirement Study; 2006 Medicare Beneficiary Annual Summary File.

Of the 4.4% of the undiagnosed sample who were classified as diabetic based on having an A1c score ≥ 6.5, only 29.9% (95% CI: 18.l-39.9) of them were reported as being diabetic in the claims data. Thus, the claims data do not differ from self-report primarily because of better classification of the undiagnosed cases.

The results are suggestive that self-reports produce a more accurate indication of a diabetes diagnosis (based on clinical data) relative to the CCW algorithm. Hence, an important question to ask is whether healthcare utilization profiles are distorted by using the CCW-algorithm to identify the diabetic population.

Table 5 shows that the healthcare utilization outcomes among discordant claims-based diabetics are generally similar to that of concordant diabetics regardless of A1c levels. However, the number of A1c tests received was markedly lower for the discordant claims-based diabetics than for the concordant diabetics (*p*-value < 0.05). This pattern of use suggests that discordant claims-based diabetics do not receive diabetes-related care at the same rate as concordant diabetics but do receive an overall level of health services and have expenditures similar to diabetics. Regression-adjusted results (controlling for demographic characteristics) yielded the same conclusions.

**Table 5:** Hemoglobin A1c levels and utilization outcomes by self-report and chronic condition warehouse diabetes status

	**Self-report, yes; Claims, yes**		**Self-report, yes; Claims, no**		**Self-report, no; Claims, yes**		**Self-report, no; Claims, no**	
Healthcare Utilization (2006)	A1c < 6.5	A1c > 6.5	A1c < 6.5	A1c > 6.5	A1c < 6.5	A1c > 6.5	A1c < 6.5	A1c > 6.5
Total reimbursement	9400.7 (1079.6)	8770.8 (1030.8)	3275.4 (1493.9)	11823.0 (8682.9)	9383.8 (1406.3)	8613.2 (2734.9)	4731.2 (320.9)	3507.0 (803.4)
Total hospitalizations	0.39 (0.05)	0.36 (0.06)	0.19 (0.10)	0.32 (0.17)	0.34 (0.06)	0.47 (0.14)	0.23 (0.02)	0.15 (0.07)
Total office visits	10.29 (0.71)	10.01 (0.52)	5.22 (1.53)	1.59 (0.41)	10.93 (0.90)	10.27 (1.86)	6.62 (0.23)	6.27 (0.73)
Total A1c tests (2002–2005)	4.67 (0.29)	4.86 (0.42)	0.48 (0.26)	0.77 (0.77)	0.78 (0.13)	2.16 (0.54)	0.09 (0.01)	0.26 (0.15)
Sample size	225	181	21	13	141	21	1377	49

Parenthetical entries are standard errors.

Source*:* 2006 Health and Retirement Study; 2006 Medicare Beneficiary Annual Summary File.

## Discussion and conclusions

In this study nationally-representative data linked with biomarker and Medicare claims were used to study the agreement between self-report and claims-based measures of diabetes. The claims-based CCW algorithm yielded a significantly higher rate of diabetics compared to self-reports. The biomarker data suggested that the higher rate of diabetics in the claims was due to false positives. False positives tended to be associated with high users of Medicare services with provider utilization profiles similar to those of concordant diabetics. Higher rates of diabetes prevalence in claims data may reflect intensive monitoring of pre-diabetic patients with elevated levels of other cardiovascular risk factors.

Our results raise potential concerns about attempts to use the current CCW diabetes indicator to identify diabetics in claims data for the purpose of assessing quality of care. Regions or accountable care organizations with high proportions of false positives may correctly fail to provide ongoing diabetes maintenance care to these patients, and thus appear to provide lower quality of care to diabetic patients (e.g., lower A1c testing).

Our findings should be interpreted in the context of the following considerations. This study relies on a single measure of Hemoglobin A1c rather than repeated measures of fasting plasma glucose or glucose tolerance test results to validate the self-report and claims-based measures of diabetes. Furthermore, people with diabetes who are controlled may have an A1c score below 6.5. We did not have access to Part D claims, though information about use of insulin or oral diabetes medications would have been another way to identify patients being treated for diabetes. However, the tradeoff would be a less representative sample as not all Medicare beneficiaries are in a stand-alone Part D plan that would have research data. Nevertheless, efforts to refine and validate claims-based algorithms with medication information may help to improve the use of these measures for health services research.

Diabetes is an important and costly chronic condition. Researchers interested in studying treatment and healthcare utilization outcomes associated with diabetes face a variety of measures to identify this subgroup. We find strengths and weaknesses of using self-reports and claims-based measures to identify the diabetic population. For researchers fortunate enough to have both measures available then the choice of which measure to use should be driven by the goals of the analysis (e.g., estimating prevalence, assessing quality of care, profiling aggregate levels of utilization) and sensitivity of the results obtained from both measures. However, if the CCW algorithm is the only measure available, then results should be interpreted with caution, particularly if those results pertain to diabetes prevalence and quality of care analyses.

## Competing interests

The authors declare that they have no competing interests.

## Authors’ contributions

JWS was involved in conceptualizing the study, designing the methodology, data acquisition, analysis, data interpretation, and drafting of the manuscript. DRW was involved in conceptualization of the study, data interpretation, and drafting of the manuscript. LHN was involved in conceptualizing the study, data interpretation, and drafting of the manuscript. All authors read and approved the final manuscript.

## Pre-publication history

The pre-publication history for this paper can be accessed here:

http://www.biomedcentral.com/1472-6963/14/150/prepub
